# Conducting Polymer Electrodes for Gel Electrophoresis

**DOI:** 10.1371/journal.pone.0089416

**Published:** 2014-02-19

**Authors:** Katarina Bengtsson, Sara Nilsson, Nathaniel D. Robinson

**Affiliations:** Transport and Separations Group, Department of Physics, Chemistry and Biology, Linköping University, Linköping, Sweden; University of Nebraska-Lincoln, United States of America

## Abstract

In nearly all cases, electrophoresis in gels is driven via the electrolysis of water at the electrodes, where the process consumes water and produces electrochemical by-products. We have previously demonstrated that π-conjugated polymers such as poly(3,4-ethylenedioxythiophene) (PEDOT) can be placed between traditional metal electrodes and an electrolyte to mitigate electrolysis in liquid (capillary electroosmosis/electrophoresis) systems. In this report, we extend our previous result to gel electrophoresis, and show that electrodes containing PEDOT can be used with a commercial polyacrylamide gel electrophoresis system with minimal impact to the resulting gel image or the ionic transport measured during a separation.

## Introduction

The quest for increased capacity and cost reduction drives miniaturization (and automation) of chemical analysis methods in life science and chemistry. However, many standard techniques, such as gel electrophoresis (GE) of proteins, DNA fragments, and other large molecules, do not lend themselves to straightforward miniaturization to “lab-on-a-chip” systems. In the specific case of GE, the uniformity of the gel, the resolution of the detection method, and the presence of undesired chemical reactions at the electrodes each impart limitations on how small effective separation devices can be. Advances in gel polymerization technology and the use of difference gel electrophoresis [1] (DIGE) have reduced the magnitude and consequences of spatial variations in gel properties dramatically, and improved imaging and detection methods have increased the resolution of the resulting gel images. In this paper, we begin to address the third (and less-frequently discussed) challenge - the electrochemical reactions at the electrodes - by performing a preliminary study exploring the potential for using electrochemically-active conjugated polymer electrodes instead of metal electrodes. Such polymer electrodes can eliminate undesired electrochemical products, reduce manufacturing costs, inhibit cross-contamination between gels, and prevent the consumption of the aqueous electrolyte, which is particularly important when reducing the volume of gel used in systems which are not immersed in electrolyte.

Simply put, electrophoresis requires the maintenance of a direct current (DC) or steady electric field in an electrolyte. This is traditionally accomplished through Faradaic reactions occurring at the interface where each electrode (anode and cathode) contacts the electrolyte. In most cases, the overall electrochemical reaction is the electrolysis of water, forming O_2_ gas, H^+^, and H_2_O_2_ at the anode, and H_2_ gas, and OH^−^ at the cathode. These products are all undesirable, as the gases produced effectively reduce the active electrode area, and the acid and base can negatively impact the molecules (proteins) being separated, particularly when electrophoresis of proteins in their native structure is intended. Furthermore, miniaturized devices often contain relatively small quantities of water, limiting that available for electrolysis before the device literally dries out.

This challenge can be addressed by oxidizing and reducing material that remains attached to or within the electrodes. The first class of materials most would consider includes metals such as Ag/AgCl, which are often either too reactive or otherwise incompatible with proteins and DNA fragments. However, the collection of pi-conjugated polymers developed for the printed electronics industry includes many materials that are both conductive and electrochemically active, and are an attractive alternative to metals due to their low cost, flexibility, biocompatibility, and ability to be deposited using a variety of processes.

The oxidation of most electrochemically-active pi-conjugated polymers can be represented as follows:
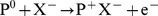
where P^0^ represents a segment of an undoped polymer chain, X^−^ represents an anion (such as Cl^−^), and e^−^ represents an electron. The oxidized polymer segment can be reduced via the reverse process.

We chose to test the suitability of a widely-used blend of poly(3,4-ethylenedioxythiophene) (PEDOT) and sodium poly(styrenesulfonate) (PSS) in the “bath-free” PHAST (TM) SDS-PAGE system from GE (here, General Electric) Healthcare Life Sciences (formerly Pharmacia). The polymer blend, denoted PEDOT:PSS, forms an aqueous emulsion that can easily be printed, coated, or cast into various forms. The negatively-charged PSS acts as the counter ion when PEDOT is oxidized (positively charged):
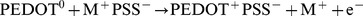
where M^+^ represents a metal cation (such as Na^+^). This type of electrochemistry is the basis for a wide variety of electronic devices, such as electrochemical transistors [2], [3], electrochromic displays [4], and bioelectronic [5] and microfluidic [6], [7] devices. PEDOT:PSS, when manufactured, contains a mixture of doped (oxidized, positively charged) and undoped (reduced, neutral) PEDOT segments, and can therefore be used for both the anode and cathode material. Note that previous (capillary) electrophoresis systems employing conjugated polymers appear to have been designed to simply use the polymer electrodes to drive electrolysis, rather than to mitigate electrolysis [8].

However, there are limitations when using π-conjugated polymer electrodes to mitigate the electrolysis of water in applications like gel electrophoresis. Most polythiophenes, including PEDOT, are susceptible to irreversible *overoxidation* when large potentials (>1 V) are applied in the presence of water and/or oxygen, particularly under basic conditions [9], [10]. Overoxidation breaks the conjugation in the polymer, rendering the polythiophene non-conductive and therefore unusable for electronic or electrochemical applications. Another limitation of oxidizing and/or reducing a pi-conjugated polymer electrode instead of electrolyzing water is the limited electrochemical capacity available. Unlike the water, which in some GE configurations is seemingly limitless, the capacity available for oxidation or reduction is a function of the size of the polymer electrode and the relative fraction of the doped and undoped material contained within. We have previously measured this capacity to be 10 C per g of “dry” PEDOT [6]. Both of these limitations can be overcome with proper device design, as demonstrated by the results presented in this work.

In addition to eliminating undesirable side-reactions at the electrodes, replacing or supplementing the fixed, expensive Pt electrodes in GE systems with electrochemically-active conducting polymer electrodes allows the electrodes to be incorporated into the gel during production via relatively inexpensive printing or coating processes. Including the electrodes in a prefabricated gel package, in turn, reduces the possibility of contamination between gels run successively in the same equipment, and facilitates reliable electronic contact between the gel and the driving electronics, enabling further miniaturization of GE systems, particularly when techniques such as DIGE help eliminate the effect of imperfections and variations within the gels themselves.

As such, in the work presented here, we have verified the compatibility of PEDOT:PSS with buffers containing sodium dodecylsulfate (SDS) and tris(hydroxymethyl)aminomethane (TRIS) and widely used in polyacrylamide gel electrophoresis (SDS-PAGE), both *ex-situ* in a simple electrochemical cell, and *in-situ*, in a real SDS-PAGE protein separation.

## Materials and Methods

We chose the PhastSystem from GE Healthcare Life Sciences, for testing our materials and techniques because it is commercially available, offers relatively easy access to the gels and electrodes (e.g., neither gel nor electrodes are submerged in a liquid buffer) and is currently used in laboratories around the world. However, we performed electronic measurements both in a PhastSystem Separation Unit and with an external measurement system based on a Keithley source-measure unit (SMU). The latter system offered considerably higher measurement resolution. The polyacrylamide (PA) gel (PhastGel 8–25, GE Healthcare) and accompanying agarose PhastGel SDS Buffer Strips (GE Healthcare), which together supply the liquid SDS buffer/electrolyte, were used in both measurement setups. Before any of these measurements, we quickly verified the ability of the PEDOT:PSS electrodes to undergo electrochemical oxidation and reduction in the SDS/TRIS electrolyte used in the PhastSystem.

### Verification of Electrochemical Compatibility between SDS/TRIS Electrolyte and PEDOT:PSS Electrodes

To quickly verify the compatibility of the SDS/TRIS electrolyte with PEDOT:PSS electrodes, we coated a standard glass microscope slide with a thin layer of PEDOT:PSS by spreading a drop of the polymer blend with the edge of a second microscope slide. After allowing the polymer film to dry, a line was scored across the width of the slide with a scalpel, creating two electronically-isolated PEDOT:PSS film electrodes on the glass slide. A thin slice of a PHAST buffer strip (agarose gel containing SDS and TRIS) was placed on top of the electrodes, bridging the gap between them and covering about 50% of each. Applying a potential of 1 V between the electrodes completed the electrochemical circuit.

### Electronic Measurements and Separation in the PhastSystem

PEDOT:PSS electrodes were fabricated by molding about 4 g of pre-baked (24 hours at 60°C) Clevios S v3 (Heraeus Precious Metals GmbH), itself a relative viscous paste, in the plastic packaging in which the SDS Buffer Strips arrive from GE Healthcare. The polymer blend was then further dried in a ventilated oven at 55°C for at least 12 hours.

Before performing experiments in the PhastSystem, the PEDOT:PSS electrodes were placed on top of a common SDS Buffer Strip, and driven at 1 V via Pt wires inserted into each electrode for 1 hour to oxidize one electrode and reduce the other. The electrodes were then placed on top of new SDS Buffer Strips in the PhastSystem, between the buffer strips, cut to 1/2 their height to accommodate the polymer electrodes, and the Pt-coated electrodes that normally contact the SDS Buffer Strips directly. The electrode that had been reduced in the first step was used as the anode, and the electrode that had been oxidized was used as the cathode. This strategy effectively doubles the capacity of the PEDOT:PSS electrodes compared to their initial capacity, when they consisted of a more-equal blend of oxidized and reduced (neutral) PEDOT. To reduce the size of the polymer electrodes required to meet the capacity requirement for an electrophoretic separation, we cut the PA gels into 11-mm-wide strips. The SDS Buffer Strips were cut to match. The current through the gel was subsequently reduced by a factor of 4 to maintain the same current density that would normally be used if the entire width of the PA gel had been used. All PhastSystem experiments were conducted at 15°C. For performing and visualizing protein separation, we used GE Healthcare’s Full Range Rainbow Recombinant Protein Molecular Weight Marker. The sample to be separated was placed on a 2 µl sample holder (GE Healthcare) in the PhastSystem. The separation was performed using the program outlined in table 1.

**Table 1:** PhastSystem program used.

Sample down at	1 Vh				
Sample up at	10 Vh				
Name of step	Maximum voltage (V)	Current (mA)	Maximum Power (W)	Temperature (°C)	Duration (Vh)
SEP 2.1 (loading step)	250	1	0.2	15	10
SEP 2.2 (separation step)	250	1	0.2	15	60

### Electronic Measurements with the Keithley SMU

**Figure 1:**
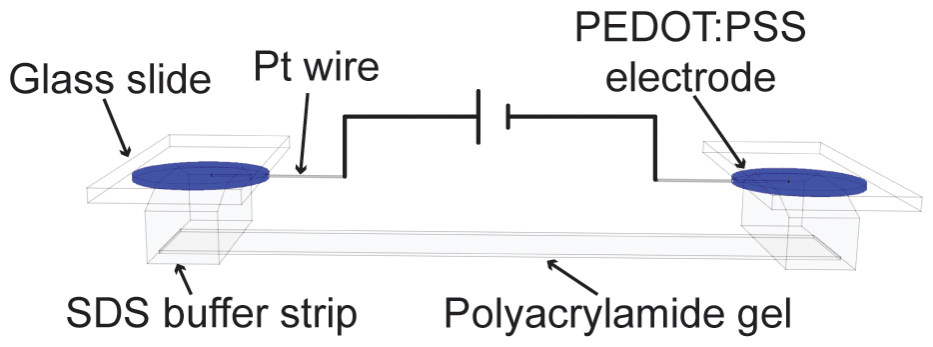
Schematic of the electrochemical cell tested with PEDOT:PSS electrodes. The Pt wires were inserted into the SDS Buffer Strips for the reference experiments.

The performance of the PEDOT:PSS electrodes was electronically measured and compared to that of Pt wire electrodes in an electrochemical cell shown schematically in figure 1. PEDOT:PSS electrodes were drop-cast onto two glass slides. A Pt wire was embedded into each electrode. An 11-mm-wide strip of the PA gel (PhastGel 8–25) was placed face-up on a lab bench. A buffer strip was placed on either end of the gel, similar to their placement during PhastSystem operation. The glass slides were placed on top of the buffer strips with the polymer electrode facing down. Connecting the Pt wire from each electrode to a Keithley 2636 source-measure unit completed the electrochemical cell, and allowed a constant potential to be applied while the current through the cell was measured.

## Results and Discussion

As described in the methods section, we built a simple electrochemical cell with two thin PEDOT:PSS electrodes on glass microscope slides connected by a SDS Buffer Strip. The PEDOT:PSS electrodes were reversibly and repeatedly oxidized and reduced by switching the polarity of an applied potential of 1 V. This was observed by a color change (electrochromism) between dark (reduced PEDOT) and light (oxidized PEDOT) blue within the electrodes, demonstrating the transport of ions between and into the electrodes, as shown in the video in the supplementary information (Video S1). Note that only the region of PEDOT:PSS contacting (under) the SDS Buffer Strip (the region directly above the silver pads used for contacting the device with the probes from the power source) is available for electrochemistry. The observed color change confirmed the compatibility of the SDS and TRIS buffer with the PEDOT:PSS, particularly that the ions are able to migrate into the partially-hydrated polymer, allowing the PEDOT through the entire thickness of the electrode to switch.

**Figure 2:**
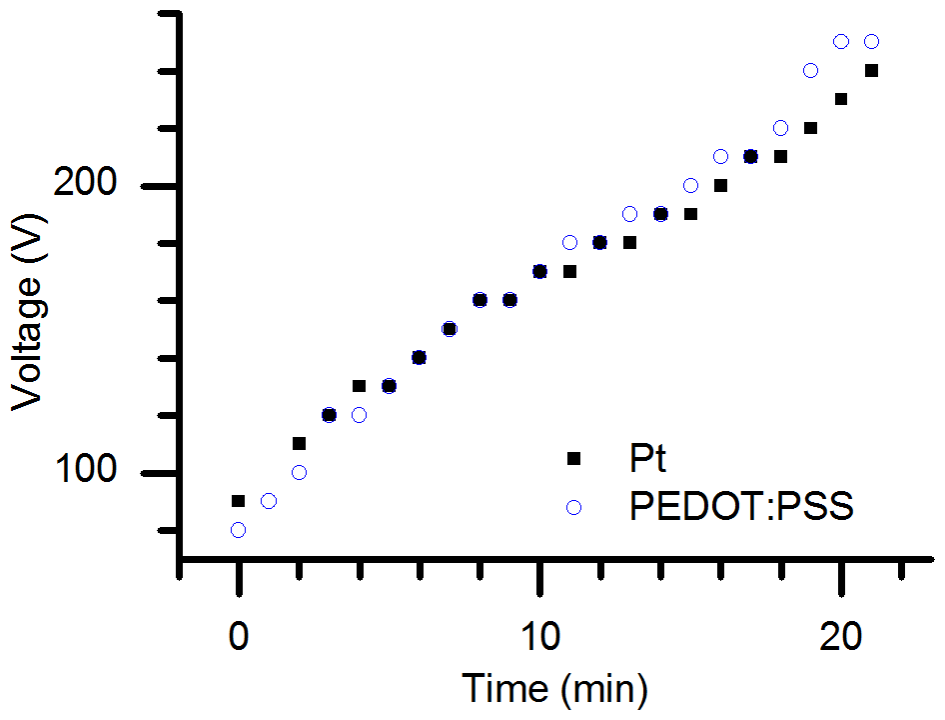
Comparison between Pt and PEDOT:PSS electrodes in the PhastSystem - Voltage. The potential applied by the PhastSystem while it maintains a constant current (1 mA, see table 1) through equivalent 11-mm-wide PA gels with Pt electrodes (solid black squares) and PEDOT:PSS electrodes (blue circles) as a function of time.

**Figure 3:**
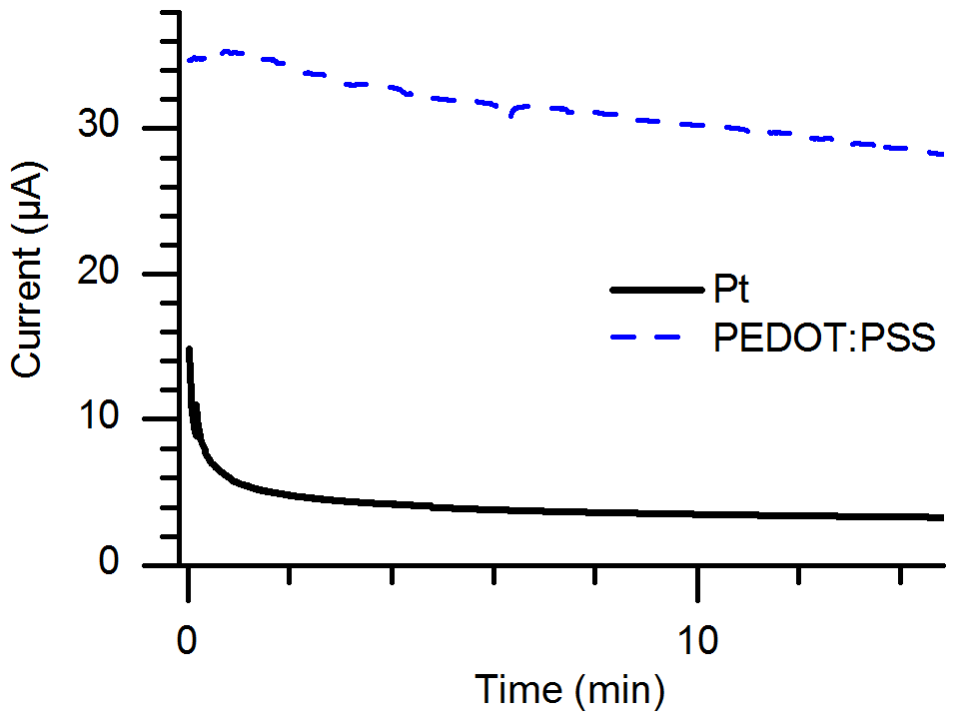
Comparison between Pt and PEDOT:PSS electrodes – current measured at 1 V. Current versus time measured through an electrochemical cell with Pt electrodes (solid black curve) and PEDOT:PSS electrodes (dashed blue curve) for an applied potential of 1 V, where water electrolysis occurs only extremely slowly in a measurement system including a dedicated source-measure unit.

**Figure 4:**
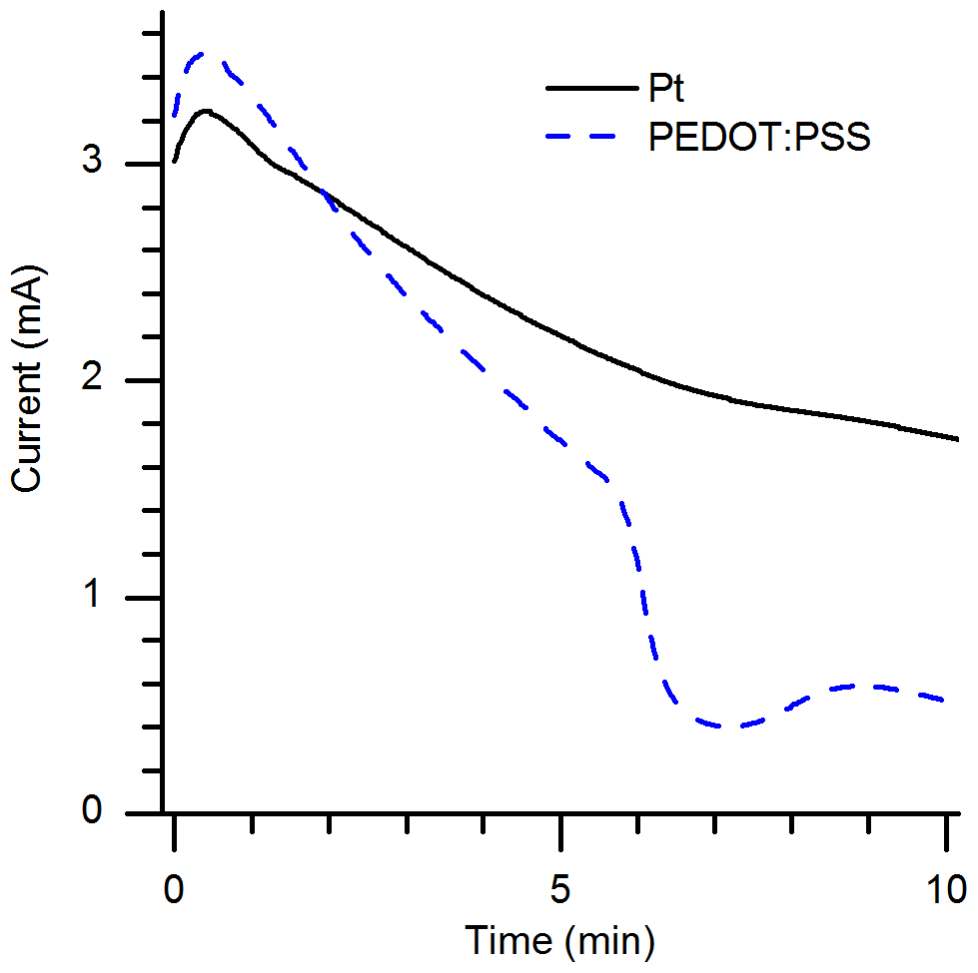
Comparison between Pt and PEDOT:PSS electrodes – current measured at 100 V. Current driven through equivalent 11-mm-wide PA gels as a function of time when 100 V was applied via Pt electrodes (solid black line) and PEDOT:PSS electrodes (dashed blue line).

Next, we compared the electronic performance of PEDOT:PSS electrodes with that of standard Pt electrodes (employing water electrolysis) in both the PHAST GE system and in a separate setup (shown in figure 1) which offered more accurate current measurement. The experiment in the PhastSystem, the results of which are shown in figure 2, demonstrates the ion-transport equivalence between gels run with Pt electrodes and PEDOT:PSS electrodes using the program described in the methods section, but at a relatively low resolution since the PhastSystem only reports current measurements to the nearest 0.2 mA. These data represent the voltage that the PhastSystem applies in order to maintain the specified current through the device (1 mA). The data from the high-resolution measurements, performed at a constant applied potential of 1 V and then 100 V using a Keithley source-meter, are shown in figures 3 and 4, respectively. For the 1 V experiment, shown in figure 3, the difference between the current through the electrochemical cell with PEDOT:PSS electrodes shows more than 6 times the current through an equivalent cell with Pt electrodes. This demonstrates that the partially-oxidized PEDOT:PSS can be further oxidized or reduced as soon as *any* potential is applied, where water electrolysis does not begin in earnest until the potential approaches 1.2 V. This assures that, even at larger applied potentials, the Faradaic reactions at the electrodes are performed on the polymer and not on water, preventing electrolytic changes in pH or gas bubble generation. See ref. [6] for more details regarding the electrochemical performance of PEDOT:PSS electrodes.

For an applied potential of 100 V, which is much closer to the conditions used in most PAGE separations, the current density through the electrochemical cells is nearly the same, as shown in figure 4. Note that the measurement using polymer electrodes shows a slightly higher current density initially, and then decreases almost linearly at a faster rate than the measurement using Pt electrodes. After about 350 seconds, the electrochemical capacity of the PEDOT:PSS electrodes is nearly fully consumed, and the current drops dramatically. This illustrates the previously-described need to design electrodes of sufficient size in any device employing the electrochemical switching of pi-conjugated polymers. Note that the electrodes used for the electrochemical measurements shown in figures 3 and 4 were taken directly from the oven, and not pre-charged using the procedure described in the Materials and Methods section.

**Figure 5:**
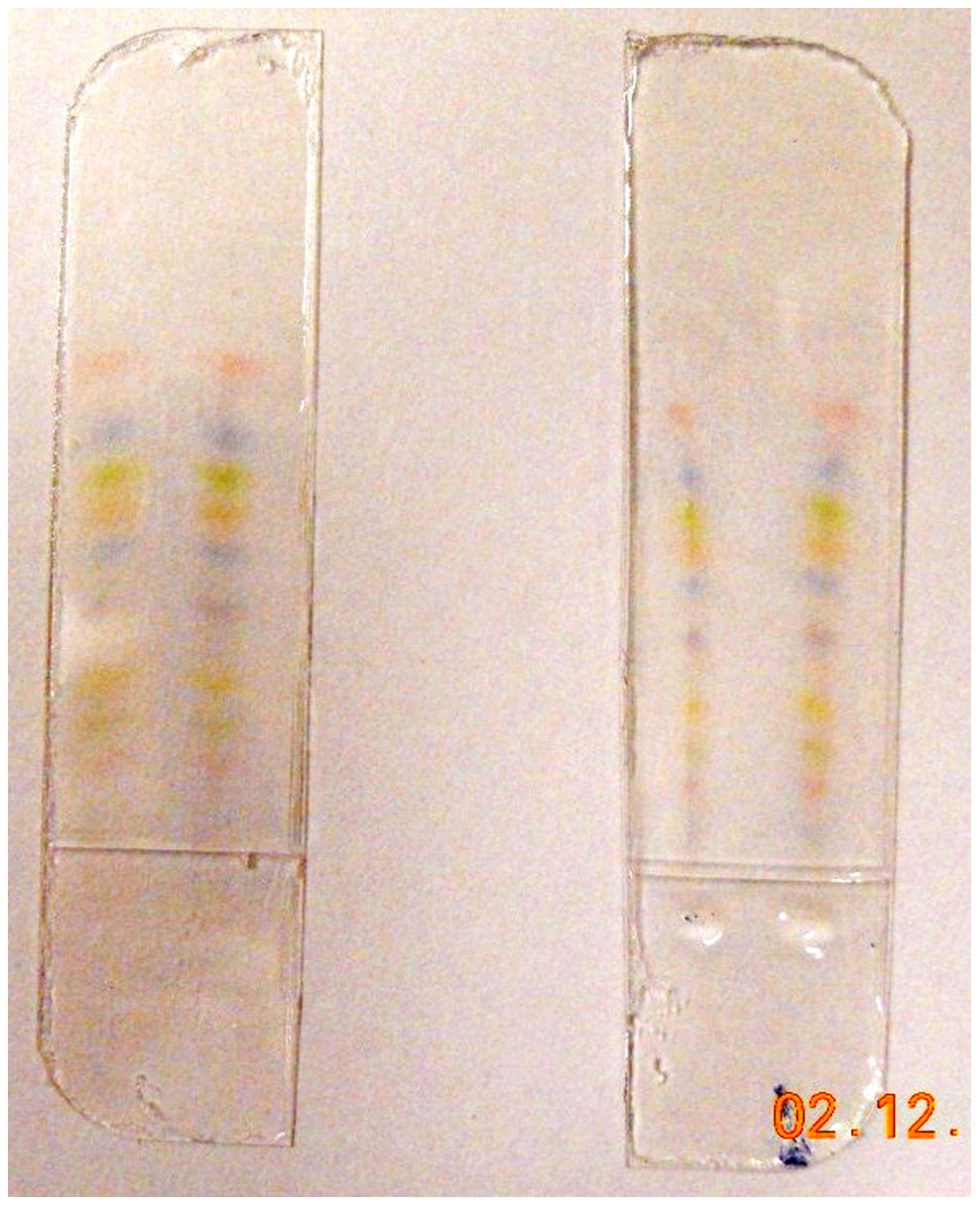
Comparison between Pt and PEDOT:PSS electrodes – gel images. Images of the Rainbow Marker separated in an 11-mm-wide PHAST PAGE gel showing the equivalence of the separation of GE Healthcare’s Full Range Rainbow Recombinant Protein Molecular Weight Marker driven with PEDOT:PSS electrodes (left) and with Pt electrodes (right). Protein migration has been driven from the cathode (top) to toward the anode (bottom) using the PhastSystem conditions shown in table 1.

We performed electrophoresis on GE Healthcare’s Full Range Rainbow Recombinant Protein Molecular Weight Marker to demonstrate the operational equivalence of the PEDOT:PSS electrodes with the standard Pt electrodes in an actual separation. Images of the resulting gels can be found in figure 5. Note that these separations were obtained during the same experiments from which the voltage vs. time data in figure 2 were collected (PhastSystem conditions shown in table 1). This experiment has been repeated at least 6 times and yielded consistent results. We verified that the PEDOT:PSS electrodes were not overoxidized during the separation in the PhastSystem by exchanging the cathode and anode and performing another separation, resulting in a similar gel image and applied voltage history.

Although the experiments above employed PEDOT:PSS electrodes in conjunction with the SDS/TRIS buffer strips sold by GE Healthcare Life Sciences, the buffer should not be necessary for maintaining the pH within the gel when electrolysis is avoided. This could require, however, the addition of SDS and electrolyte (salt) to the sample being studied.

## Conclusions

We have demonstrated that PEDOT:PSS electrodes are chemically and electrochemically compatible with SDS-PAGE separations via electrophoresis of a standard benchmark protein mixture. Aside from introducing the polymer electrodes, no change is required in the protocol for performing SDS-PAGE. This result, in conjunction with our previous demonstration of the reduction of water electrolysis when using PEDOT:PSS electrodes [6], has the potential to pave the way for the development of low-cost, disposable, miniaturized GE systems for accelerated analysis in areas such as proteomics and medical diagnosis (e.g., analysis of proteins associated with tumors in bodily fluids). We hope that this will lead to more advanced and less expensive diagnoses in modern healthcare facilities and in areas of the world where advanced laboratory analyses are not yet readily available.

## Supporting Information

Video S1 **Video showing electrochromic switching in two PEDOT:PSS electrodes connected by a piece of** **PhastGel SDS Buffer Strips.** The PEDOT:PSS electrodes were reversibly and repeatedly oxidized and reduced by switching the polarity of an applied potential of 1 V. This was observed by a color change (electrochromism) between dark (reduced PEDOT) and light (oxidized PEDOT) blue within the electrodes, demonstrating the transport of ions between and into the electrodes. Only the region of PEDOT:PSS contacting (under) the SDS Buffer Strip (the region directly above the silver pads used for contacting the device with the probes from the power source) is available for electrochemistry. The observed color change confirmed the compatibility of the SDS and TRIS buffer with the PEDOT:PSS, particularly that the ions are able to migrate into the partially-hydrated polymer, allowing the PEDOT through the entire thickness of the electrode to switch.
(MP4)
